# Global 5-Hydroxymethylcytosine Levels Are Profoundly Reduced in Multiple Genitourinary Malignancies

**DOI:** 10.1371/journal.pone.0146302

**Published:** 2016-01-19

**Authors:** Enrico Munari, Alcides Chaux, Ajay M. Vaghasia, Diana Taheri, Sarah Karram, Stephania M. Bezerra, Nilda Gonzalez Roibon, William G. Nelson, Srinivasan Yegnasubramanian, George J. Netto, Michael C. Haffner

**Affiliations:** 1 Department of Pathology, Johns Hopkins University, Baltimore, Maryland, 21231, United States of America; 2 Sidney Kimmel Comprehensive Cancer Center, Johns Hopkins University, Baltimore, Maryland, 21231, United States of America; 3 Brady Urological Institute, Johns Hopkins University, Baltimore, Maryland, 21231, United States of America; 4 Department of Scientific Research, Norte University; Centro para el Desarrollo de la Investigación Científica (CEDIC) Asunción, Asunción, Paraguay; 5 Department of Pathology, Isfahan University of Medical Sciences, Isfahan Kidney Diseases Research Center, Isfahan, Iran; Innsbruck Medical University, AUSTRIA

## Abstract

Solid tumors are characterized by a plethora of epigenetic changes. In particular, patterns methylation of cytosines at the 5-position (5mC) in the context of CpGs are frequently altered in tumors. Recent evidence suggests that 5mC can get converted to 5-hydroxylmethylcytosine (5hmC) in an enzymatic process involving ten eleven translocation (TET) protein family members, and this process appears to be important in facilitating plasticity of cytosine methylation. Here we evaluated the global levels of 5hmC using a validated immunohistochemical staining method in a large series of clear cell renal cell carcinoma (n = 111), urothelial cell carcinoma (n = 55) and testicular germ cell tumors (n = 84) and matched adjacent benign tissues. Whereas tumor-adjacent benign tissues were mostly characterized by high levels of 5hmC, renal cell carcinoma and urothelial cell carcinoma showed dramatically reduced staining for 5hmC. 5hmC levels were low in both primary tumors and metastases of clear cell renal cell carcinoma and showed no association with disease outcomes. In normal testis, robust 5hmC staining was only observed in stroma and Sertoli cells. Seminoma showed greatly reduced 5hmC immunolabeling, whereas differentiated teratoma, embryonal and yolk sack tumors exhibited high 5hmC levels. The substantial tumor specific loss of 5hmC, particularly in clear cell renal cell carcinoma and urothelial cell carcinoma, suggests that alterations in pathways involved in establishing and maintaining 5hmC levels might be very common in cancer and could potentially be exploited for diagnosis and treatment.

## Introduction

Epigenetic alterations are amongst the most common changes in human malignancies. [1–4] In particular changes in the pattern of cytosine methylation have been extensively studied in numerous cancer types and show favorable properties as disease biomarkers. [1,4,5] Methylation of the C5-position of cytosines (5mC) within a CpG context, in particular in regions of high CpG density (CpG islands), is often associated with transcriptional repression. In general, in many solid tumors, the global levels of 5mC show a decrease whereas methylation marks are gained in particular sites of the genome establishing stable and heritable alterations that can contribute to cancer progression. [2,3] Despite the stability of these marks, there is evidence for ongoing epigenetic plasticity and clonal selection in solid tumors. [6–8] A particularly intriguing potential mechanism for dynamic alterations in the 5mC genomic content is the oxidation of 5mC to 5-hydroxymethylcytosine (5hmC). 5hmC was first characterized in bacteriophages and was later also detected in mammalian genomes. [9] More recently, the observation that 5mC can become oxidized in a highly directed manner to 5hmC by the ten eleven translocation protein family (TET1-3) has spurred increased interest in 5hmC biology. [10–13] Importantly, it was shown that 5hmC can be further metabolized by TET enzymes to 5-formylcytosine and 5-carboxycytosine, which can subsequently be removed from the genome by base excision repair or decarboxylation. [14,15] Therefore, on one hand the oxidation of 5mC to 5hmC represents an intriguing pathway for epigenetic plasticity. On the other hand it is important to note that many tissues accumulate high levels of 5hmC, exceeding thresholds that would be expected from a simple intermediate, suggesting that 5hmC could be an independent epigenetic mark with unique signaling properties. The potential signal transduction mechanisms of 5hmC have not been fully elucidated; however distribution pattern of 5hmC in mammalian genomes suggest a role of 5hmC for transcriptional regulation, since 5hmC marks have been shown to be most prevalent at upstream regulatory gene regions. [12,16,17]

Our group has recently developed a robust immunohistochemical staining protocol that enables the *in situ* evaluation of global 5hmC levels on a cell-by-cell basis. [18] Intriguingly, global 5hmC have been shown to be tightly correlated with differentiation in normal hierarchically organized tissues, in which tissue stem cell compartments show very low levels of 5hmC whereas differentiated cells are characterized by high levels of 5hmC. In addition, global levels of 5hmC have been shown to be profoundly decreased in solid tumors. [18–27]

Given this demonstrated cancer-specific loss of 5hmC, robust antibody based detection systems could be used as a diagnostic aid in multiple malignancies. To evaluate the distribution and cancer specific loss of 5hmC in urological malignancies, we stained a large series of urothelial cell carcinoma of the bladder, renal cell carcinoma and testicular germ cell tumors and correlated staining results with histopathologcial parameters and clinical outcome data.

## Materials and Methods

### Ethics Statement

Tumor and adjacent normal tissue samples were obtained from the archives of the Johns Hopkins Hospital Department of Pathology following appropriate institutional review board approval. No informed consent (verbal or written) was obtained from the retrospective tissue specimens. The research ethics committee waived the requirement for informed consent for samples included in the tissue microarrays. The patient data was anonymized prior to use in the study.

### Patient Cohorts and Tissue Samples

Tissue samples from 111 patients with clear cell renal cell carcinoma (CCRCC) (Table 1), 55 patients with urothelial carcinoma of the bladder (UC) (Table 2) and 84 with testicular germ cell tumors (TGCT) (Table 3) treated at Johns Hopkins Medical Institutions (Baltimore, MD) were included. All archival tissues were reviewed by an expert urological pathologist (G.J.N.) for confirmation of the original diagnosis in accordance with the American Joint Committee on Cancer Classification, 7^th^ Edition 2010. Using a previously described procedure [28], 4 sets of tissue microarrays (TMA) were constructed: the first contained 70 selected samples of primary CCRCC treated by either partial [39] or radical nephrectomy [31] in the year 2005; the second set included non-matched metastatic CCRCC diagnosed between 2004 and 2006. The third included 55 patients treated by cystectomy with a diagnosis of UC between 1994 and 2002. The fourth set contained 60 pure TGCT (including 47 seminomas, 6 embryonal carcinomas, 3 yolk sac tumors, 3 teratomas and 1 spermatocytic seminoma) and 24 mixed TGCT treated by orchiectomy between 1995 and 2008. Each tumor sample was represented by at least 3 independent tissue cores. Adjacent benign tissue was included for every case and served as an internal control. In primary CCRCC and UC, pelvic recurrence and metastatic disease to distant sites were considered as indicative of tumor progression. For TGCT, metastasis to lymph nodes or distant sites was considered as tumor progression.

**Table 1:** Clinicopathologic characteristics of primary CCRCC cohort.

Parameter	No. of cases (%)
**Age at nephrectomy (y)**	
Mean	54.6
Median	55 (22–82)
**Sex (%)**	
Male	51 (73)
Female	19 (27)
**Pathologic stage at nephrectomy**	
pT1	50/69 (73)
pT2	2/69 (3)
pT3	17/69 (24)
pT4	0
**Ethnicity (%)**	
Caucasian	54 (77)
African American	12 (17)
Other	4 (6)
**Progression (%)**	4/69 (6)

**Table 2:** Clinicopathologic characteristics of bladder cancer cohort.

Parameter	No. of cases (%)
**Age at cystectomy (y)**	
Mean	65.3
Median	67 (34–88)
**Sex (%)**	
Male	44 (80)
Female	11 (20)
**Pathologic stage at nephrectomy**	
pTa	2 (4)
pTis	4 (7)
pT1	5 (9)
pT2	17 (31)
pT3	21 (38)
pT4	6 (11)
**Ethnicity (%)**	
Caucasian	50 (91)
African American	5 (9)
**Progression (%)**	29/43 (67)

**Table 3:** Clinicopathologic characteristics of the testicular germ cell tumor (TGCT) cohort.

Parameter	No. of cases (%)
**Age at orchiectomy (y)**	
Mean	31.7
Median	30.5 (1–67)
**Pathologic stage at orchiectomy**	
pT1	52/84 (62)
pT2	24/84 (29)
pT3	7/84 (8)
pT4	1/84 (1)
**Ethnicity (%)**	
Caucasian	69/84 (82)
African American	6/84 (7)
Other	9/84 (11)
**Progression (%)**	6/62 (9.7)

### Immunohistochemistry and Immunofluorescence Staining

Immunolabeling of 5hmC was performed as described previously. [18] In brief, 5 micron paraffin sections were de-waxed and rehydrated following standard protocols. Antigen retrieval consisted of steaming for 30 min in citrate buffer (pH 6.0) followed by incubation in 3.5 N HCl for 15 min at room temperature. For immunolabeling of 5hmC, a rabbit polyclonal 5 hydroxymethylcytosine specific antibody (Active Motif, Cat # 39769, Carlsbad, CA) was applied at 1:20,000 dilution for 1 h at room temperature. DNMT1 staining was performed using previously published protocols [29]. Immuncomplexes were detected using the PowerVision+™ immunohistochemistry detection system from ImmunoVision Technologies (Norwell, MA, USA) with 3,3′-diaminobenzidine tetrahydrochloride (DAB) as the chromogen. For double immunofluorescence labeling of 5hmC and ki67 pretreatment conditions were identical to chromogenic staining. 5hmC and ki67 (Zymo Research) antibodies were used at 1:6000 and 1:100 dilutions respectively. Immuncomplexes were visualized with Alexa 488 nm anti-rabbit and Alexa 565 nm anti-mouse (Life Technologies, Grand Island, NY) secondary antibodies. After nuclear counterstaining with DAPI, slides were cover slipped with Prolong (Life Technologies). All slides were imaged on a Nikon 50i epifluorescence microscope. Immunofluorescence images were captured using a CoolsnapEZ digital camera (Photometrics, Tucson, AZ) and the Nikon NIS-Elements (Nikon, Melville, NY) software package.

### Scoring System

For each case, positive immunoreactivity was first evaluated in adjacent normal tissue. Only cores that showed robust immunostaining in adjacent benign stromal cell nuclei were evaluated. Uniformly, immunoreactivity was restricted to the nucleus. To evaluate not only the percentage of positive cells but also the staining intensity in the positive cells, we employed an H-score system, which was assigned to each TMA spot as the sum of the products of intensity of staining (0 for negative, 1 for weakly positive, 2 for moderately positive and 3 for strongly positive) by the extent of immunoexpression (0–100), obtaining a value from 0 to 300 (representative case and corresponding H-scores are shown in **S1 Fig**). Final H-scores for each case were obtained as the average of all the individual H-scores of each TMA spot, and these were subsequently used in statistical analyses.

### Statistical Analysis

Association between variables were evaluated using the Mann-Whitney U/Kruskal-Wallis test and the Fisher's exact test. For statistical analysis 5hmC expression was categorized in low and high levels using the upper tertile as the cutoff point. Hazard ratios for predicting overall mortality, cancer-related mortality, metastatic disease, and tumor progression were estimated using Cox's proportional hazards regression. Hazard ratios were also adjusted by clinicopathologic features. Correlation between biomarker levels were estimated using the Spearman rank correlation test (rho coefficient). A 2-tailed P < 0.05 was required for statistical significance. Data were analyzed using R Version 3.1.1 “Sock it to Me” (R Foundation for Statistical Computing, Vienna, Austria).

## Results

To evaluate the global levels of 5hmC in a broad spectrum of urological malignancies, we used a previously validated immunohistochemical staining protocol and determined global 5hmC levels in a representative cohort of urothelial cell carcinoma of the bladder (UC) (n = 55), clear cell renal cell carcinoma (CCRCC, primary carcinoma n = 70, metastases n = 41) and testicular germ cell tumors (TGCT, n = 84) and adjacent benign tissues.

**Fig 1:**
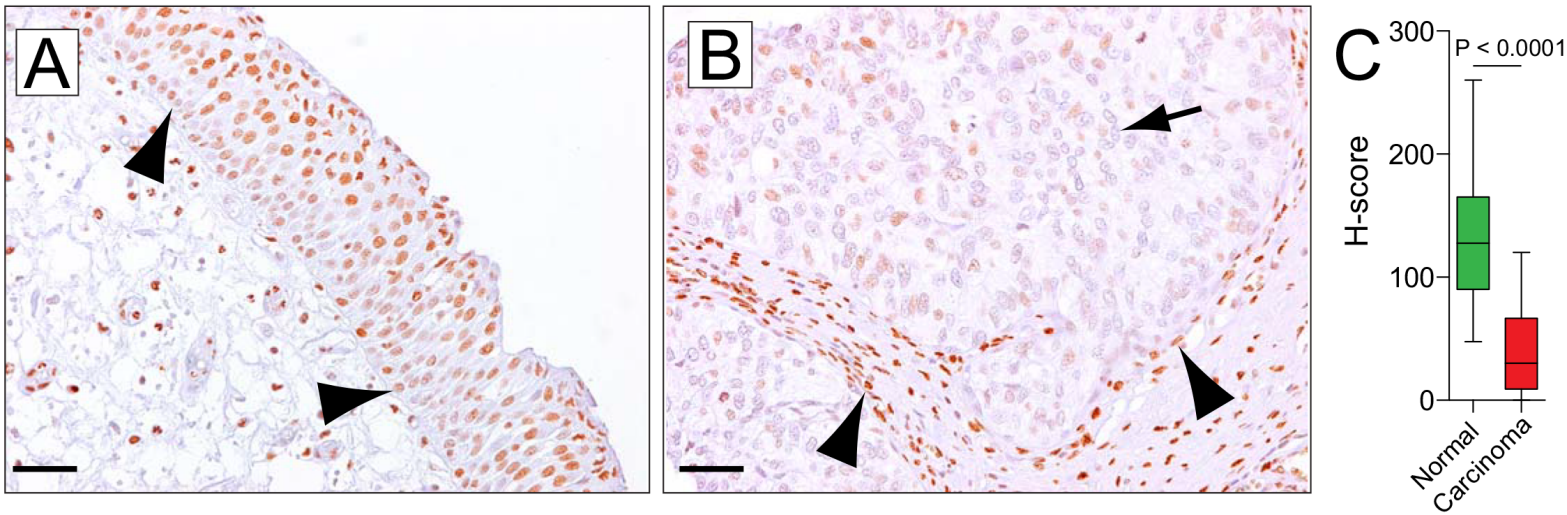
Urothelial carcinoma of the bladder shows low 5hmC levels. (A) Normal transitional cell epithelium of the bladder shows strong nuclear 5hmC staining in apical cell layers; basal cell layers exhibit reduced staining intensities (arrowhead). (B) Urothelial carcinoma shows reduced nuclear 5hmC (arrow). Note that tumor-associated stromal cells show robust strong staining and function as an internal staining control. (C) Boxplots depicting H-score distribution in normal urothelium and urothelial carcinoma. All images were taken at original magnification of 200x. Scale bars indicate 50 μm.

Normal urothelium showed robust nuclear immunolabeling for 5hmC. As shown previously, cells in the basal cell layer showed reduced 5hmC staining (**Fig 1A**). [18] The staining distribution of 5hmC showed no direct association with cell proliferation as measured by ki67 staining (**S2 Fig, S6 Fig, S10 Fig**). Consistently, strong nuclear staining was also observed in associated stromal cell nuclei. Invasive carcinoma (n = 55) showed overall greatly reduced 5hmC levels (mean = 107 and mean = 30 for normal urothelium and UC respectively, P < 0.0001, **Fig 1B and 1C**). No difference was observed between superficial and invasive lesions, suggesting that 5hmC loss could represent an early event in cancer progression (**S3 Fig**). 5hmC levels were not associated with stage (P = 0.19). There was a trend, albeit not statistically significant for tumors with higher 5hmC H-scores to show lower incidence of lymph node metastases (0% vs 24%, P = 0.17). However, Cox-proportional hazard regression models failed to show a prognostic significance of 5hmC levels in unadjusted or adjusted models for predicting overall mortality (HR = 1.2, P = 0.62 and HR = 0.98, P = 0.96, respectively) and cancer-related mortality (HR = 1.5, P = 0.48; HR = 0.69, P = 0.59, respectively) (**S1 Table and S4 Fig**).

**Fig 2:**
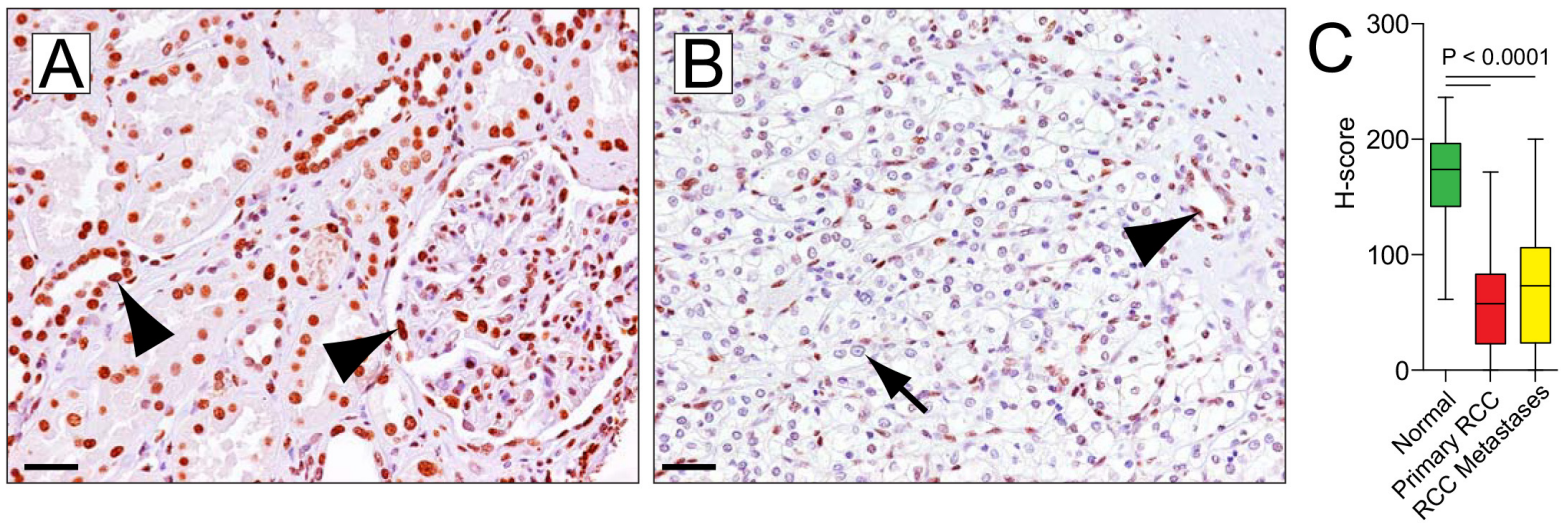
Global loss of 5hmC in primary renal cell carcinoma and metastases. (A) Normal kidney tissue shows uniformly strong staining for 5hmC in tubules and glomerular cells. (B) Clear cell renal cell carcinoma exhibit greatly reduced 5hmC levels (arrows). Note that stromal and endothelial cells show labeling with 5hmC antibodies. (C) Boxplots illustrating H-score distribution in normal kidney tissue, primary renal cell carcinoma and metastases. All images were taken at original magnification of 200x. Scale bars indicate 50 μm.

Normal kidney showed nuclear 5hmC in all cell types with strong staining in tubules and glomeruli (**Fig 2A**). Primary CCRCC (n = 70) showed reduced 5hmC levels in the cancer cell nuclei (mean H-scores 168 and 60 for normal kidney and CCRCC respectively, P < 0.0001; **Fig 2B and 2C**). Tumor associated stroma cells showed robust staining (**Fig 2B**). Higher grade lesions showed lower 5hmC levels (**S6 Fig)** and a trend towards higher stage tumors was observed in lesions with lower 5hmC H-scores (P = 0.08). Similar to primary CCRCC (mean = 60), distant renal cell carcinoma metastases showed reduced 5hmC levels (mean = 74), significantly different from normal epithelium (mean = 168, P < 0.0001) (**Fig 2C, S6 Fig**). There was no difference in 5hmC levels between primary and metastatic CCRCC (P = 0.21). Furthermore, in the limited number of patients that showed tumor progression during the observation period, no statistically significant association between tumor 5hmC levels and mortality was observed (HR = 0.48, P = 0.39) (**S2 Table and S7 Fig**). Interestingly, 5hmC levels were positively correlated with p27, PTEN and HIF expression levels (rho = 0.42, P = 0.0001; rho = 0.33, P < 0.0001; and rho = 0.63, P < 0.0001, respectively) determined in a previous study published from our group (**S8 Fig**). [30] Across different tumor types, no correlation between 5hmC and DNMT1 levels was observed (**S8 Fig**).

**Fig 3:**
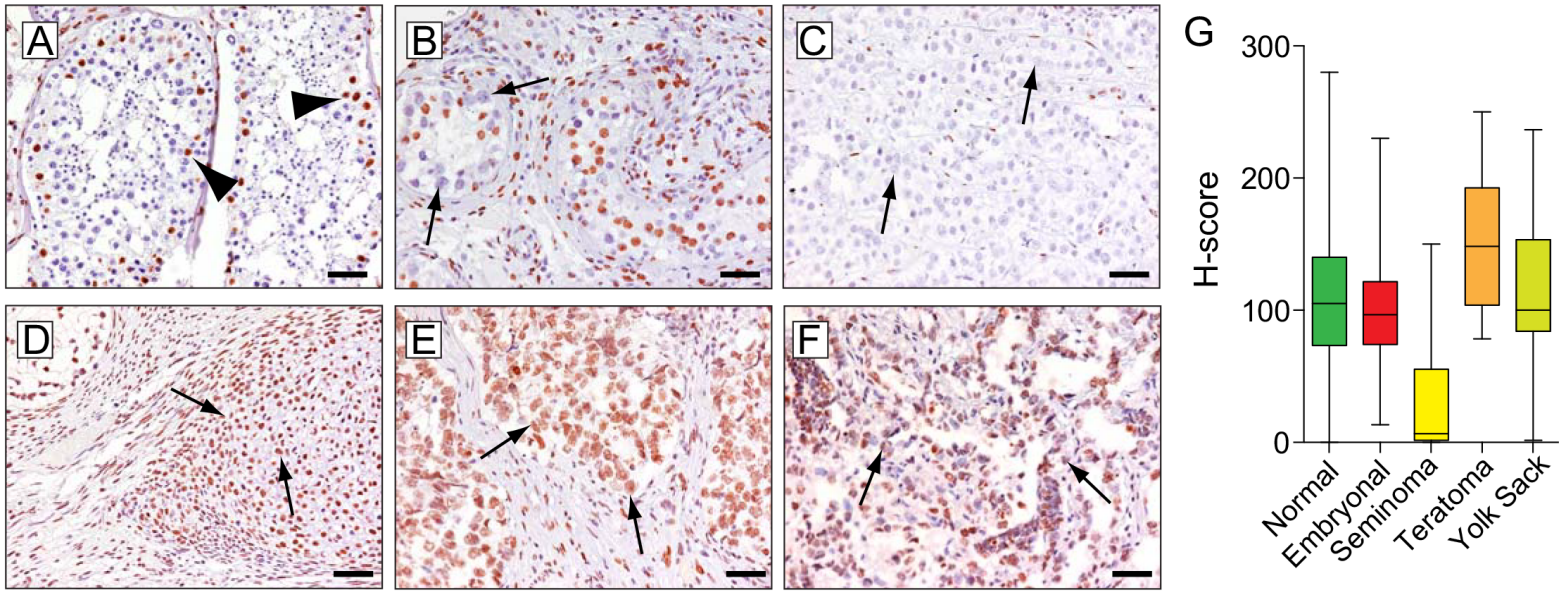
Global 5hmC levels in normal testis and testicular neoplasms. (A) Sertoli cells (arrowheads) show strong immunoreactivity in normal testicular tubules. (B) Low 5hmC staining in intratubular germ cell neoplasia (ITGCN) of the testis (arrows). (C) Representative seminoma case with low nuclear 5hmC staining. (D) Representative mature teratoma exhibiting robust nuclear staining. Representative embryonal carcinoma (E) and yolk sack tumor (F) exhibiting variable degrees of 5hmC staining levels in neoplastic cell nuclei. (G) Boxplots summarizing staining distribution in testicular germ cell tumors (seminoma n = 48; embryonal carcinoma; n = 24, yolk sac tumor n = 16; teratoma n = 16) (TGCT) lesions. Note that in tumors with mixed morphologies, components were scored separately. All images were taken at original magnification of 200x. Scale bars indicate 50 μm.

Normal testis showed low levels of nuclear staining in spermatogonia and differentiated spermatids. Sertoli cells and stromal cells outside of the seminiferous tubules showed strong immunoreactivity for 5hmC (**Fig 3A**). Note that intratubular neoplastic cells (ITGCN) were characterized by absence of 5hmC staining (**Fig 3B, arrows**). Seminoma showed uniformly greatly reduced nuclear 5hmC staining (mean: 30)(**Fig 3C**). Teratoma, in particular mature areas showed very high levels of 5hmC (mean: 93), whereas yolk sack tumors and embryonal carcinoma showed intermediate median staining intensities with wide distribution ranges (mean: 78 and 53, respectively) (**Fig 3G**).

## Discussion

The recent discovery of enzymes that can convert 5mC to 5hmC has greatly broadened our understanding about the complexities of epigenetic regulation. [10,12] Importantly, in contrast to global 5mC levels it was demonstrated that levels of 5hmC vary significantly in different cell states. [11,18,31,32] Tissue stem cells and cells in the proliferating cell compartment, in both hierarchically organized epithelia and in the hematopoietic system were characterized by overall low levels of global 5hmC. [18,19] Terminally differentiated cells on the other hand showed high levels of 5hmC in almost all cell lineages evaluated thus far. [18,33] These observations suggest that 5hmC is tightly linked to cellular differentiation. Interestingly, it was also noted that most solid tumors are characterized by a profound global loss of 5hmC with striking differences between invasive carcinoma and adjacent normal tissue. [18,20,21] Previous studies have evaluated several tumor types including breast, prostate and colon carcinoma, but no detailed information on the levels of 5hmC in urological malignancies including CCRCC, UC and TGCT is available. Given the potential of 5hmC as a highly tumor specific marker, we evaluated 5hmC levels in a representative cohort of urological malignancies.

We found that both UC and CCRCC showed greatly reduced 5hmC levels compared to adjacent benign tissues, suggesting that the tumor specific loss of 5hmC could eventually be of diagnostic utility if further validated. It is however worth noting that the degree of loss in both tumor entities was not strongly correlated with grade or stage and showed no association with prognosis. This is in contrast to previous observations in malignant glioma and melanoma, where 5hmC levels were negatively correlated with tumor grade and showed prognostic significance. [19,23] It is however worth noting that the present study was not adequately powered to detect small prognostic differences based on 5hmC levels, since the number of progression and disease specific death events were overall low. Despite the profound global loss of 5hmC in both CCRCC and UC, a certain level of staining heterogeneity with low level staining in a subset of cancer cell nuclei (**Figs 1 and 2**) was noted within individual tumor lesions. This heterogeneity would argue against a passive loss of 5hmC and would more likely suggest that other processes maintaining 5hmC integrity are disrupted in neoplastic cell.

An intriguing explanation for the global loss of 5hmC in neoplastic cells is the functional perturbation of TET enzyme. TET1-3 are involved in oxidation of 5mC to 5hmC and have been shown to be corrupted in tumors on multiple levels. For instance, mutations in TET2 have been observed in numerous malignancies [34], expression level of TET enzymes are greatly reduced in some cancers [35], oncometabolites (such as 2-hydroxyglutarate) can result in inhibition of TET function, [36] and cytoplasmic sequestration of TET enzymes can result in decreased locally active enzyme levels. [37] In particular the presence of 2-hydroxyglutarate could be relevant in renal cell carcinoma, since a recent study demonstrated that 2-hydroxyglutarate is present at high abundance in a subset of RCC and is associated with lower global 5hmC levels. [38] In addition, other enzymes involved in the metabolism of 5hmC can be altered in cancer. For instance, DNMTs (in particular DNMT3A and DNMT3B), which can potentially convert 5hmC to C, are frequently overexpressed in cancer. [39] It is worth noting that in this study we did not observe an association between DNMT1 and 5hmC levels, suggesting that either a transient imbalance in DNMT1 expression can lead to cancer specific 5hmC loss or, more likely additional enzymes involved in 5hmC metabolism are altered. For instance, Thymine DNA Glycosylase (TDG), which can deaminate 5hmC and therefore contribute to the reduction of the 5hmC pool, is also dysregulated in cancer cells. Given the complex biology of 5hmC and associated epigenetic changes it remains to be shown if changes in 5hmC itself as an instructive epigenetic mark or rather other cellular perturbation that lead to passive genome wide loss of 5hmC contribute to the malignant phenotype.

It is noteworthy that changes in 5hmC levels were restricted to neoplastic cells while tumor associated stroma as well as adjacent histologically benign tissue showed robustly high 5hmC levels. This contrast in 5hmC staining levels between neoplastic and non-neoplastic tissue suggests that 5hmC could be of potential utility as a cancer detection biomarker. Importantly, both invasive as well as superficial UC show similar 5hmC levels, suggesting that loss of 5hmC could be an early event during carcinogenesis. Furthermore, 5hmC levels in metastases from CCRCC and UC were similar to the primary tumor, further supporting that changes in 5hmC levels likely occur in the early phases of neoplastic transformation and do not change significantly during tumor dissemination.

Interestingly a positive correlation between p27, PTEN and 5hmC was observed, indicating that in cells that are proficient of the endogenous Akt and CDK inhibitor PTEN and p27 5hmC levels are high. This correlative observation provides evidence for link between PI3K signaling and 5hmC biology and suggests that cells undergoing cell cycle arrest (high p27) show higher 5hmC levels. Furthermore, HIF1-α levels were also correlated with 5hmC possibly implying that changes in the HIF1 signaling axis could be involved in epigenetic regulation. [40] This finding could also be interpreted as an indicator of the complex relationship between oxygen tension and 5hmC biology. In this context it is worth noting that molecular oxygen, together with alpha-ketoglutarate is required for the oxidation of 5mC to 5hmC by the tet-protein family.

TGCTs showed a broad spectrum of 5hmC levels. Whereas seminoma exhibited very low levels of 5hmC, mature teratoma showed robust staining for 5hmC, recapitulating the staining pattern observed in normal differentiated tissue equivalents. More generally, stem cells in the proliferating cell compartment of the gastrointestinal tract and hematopoietic system show low levels of 5hmC. This could suggest that in some tissue types proliferation and lack of adequate maintenance of 5hmC could result in a passive loss of 5hmC over time. [41,42] It is worth noting that in the present study, we did not find a strong correlation between 5hmC levels and cell proliferation, as measured by ki67 co-immunolabeling. Interestingly, global 5hmC content decreases rapidly as cells from normal tissue adapt to cell culture [43]. On the other hand, normal epithelial cell lines can be reconverted to showing high levels of 5hmC by allowing them to differentiate in 3D culture systems [44], suggesting that despite active proliferation, 5hmC can be maintained at high levels in the appropriate cellular context. These findings suggest that differentiation even in the background of oncogenically transformed cells is associated with increased 5hmC levels. The low level of 5hmC in seminoma is in line with previous observations suggesting that during spermatogenesis 5hmC decreases while 5mC remains constant. [26,29] In general seminomas share characteristics of primordial germ cells, whereas embryonal carcinoma are considered the malignant counterpart of embryonal stem cells. This difference in the cell of origin, with different epigenetic states, might explain some of the differences in 5hmC levels observed here. Along these lines, the low levels of 5hmC may reflect the very low levels of 5mC previously observed in seminomas. Furthermore, in agreement with previous observations, we also found that ITGCN cells showed low levels of 5hmC. [26,45]

In conclusion, this study establishes that global 5hmC levels are greatly reduced in the majority of urological malignancies. This is important from both a basic biology but also clinical diagnostic perspective. The uniform loss of 5hmC suggests a unique mechanism for 5hmC maintenance that is corrupted in neoplastic cells of different cell lineages. Ongoing mechanistic studies, as well a detailed genome wide mapping efforts will help shed more light on 5hmC disease biology. [36,38,46] From a diagnostic standpoint, additional detailed studies are needed to address the usefulness of this biomarker.

## Supporting Information

S1 Dataset (XLSX)

S1 Fig Representative images and H-scores of (A) urothelial carcinoma, (B) clear cell renal cell carcinoma and (C) testicular germ cell tumors stained for 5hmC. (PDF)

S2 Fig Co-immunolabeling of 5hmC and ki67 in normal bladder and urothelial carcinoma of the bladder. (A) Co-immunolabeling of 5hmC (red) and ki67 (green) in normal urothelium shows low ki67 labeling (mostly localized in the intermediate cell layer) and a gradual increase of 5hmC with increased distance from the basal cell layer. (B) In urothelial carcinoma 5hmC levels are uniformly reduced. Ki67 labeling is present in a large fraction of cells (arrows). No difference in 5hmC immunoreactivity is detected between ki67 positive and ki67 negative cells.
(PDF)

S3 Fig 5hmC levels in invasive and superficial urothelial cell carcinoma of the bladder. **(**A) Box plot shows 5hmC H-score distribution of tumor and normal urothelium. (B) H-score distribution in invasive and non-invasive urothelial cell carcinoma. (C) Representative micrographs of invasive urothelial carcinoma of the baldder stained for 5hmC and p53. (D) Representative micrographs of non-invasive urothelial carcinoma of the baldder stained for 5hmC and p53. Note that Invasive carcinoma show nuclear accumulation of p53 suggestive of mutant *TP53*. 5hmC levels are not different between invasive and non-invasive carcinoma.
(PDF)

S4 Fig Association of global 5hmC levels with clinicopathological characteristics in urothelial carcinoma. **(**A) Box plot showing median H-score distribution between normal urothelium and urothelial cell carcinoma. (B) Box plot show 5hmC level differences between specimens with and without progression to metastasis. (C) Box plot showing 5hmC levels in lesions with and without synchronous lymph node metastasis. (D) Box plot showing 5hmC levels between specimens with and without tumor progression. (E) Box plot showing 5hmC levels based on cancer specific survival. (F) Lymph node metastasis at the time of surgery is a strong predictor for disease specific and overall mortality. (G) 5hmC levels are not associated with cancer related mortality. P-values for boxplot comparisons by Wilcoxon rank test. P-values from Kaplan-Meier analysis are log-rank test derived.
(PDF)

S5 Fig Co-immunolabeling of 5hmC and ki67 in (A) normal kidney and (B) clear cell renal cell carcinoma. Note that 5hmC levels are high in normal kidney tissue and greatly reduced in renal cell carcinoma. Sporadic ki67 positive cells (stained in green) can be found in carcinoma. Note that no direct association between ki67 positive cells and 5hmC staining was observed.
(PDF)

S6 Fig 5hmC levels in normal kidney, primary renal cell carcinoma and metastatic renal cell carcinoma. **(**A) Density plot shows distribution of 5hmC levels in normal tissue (red) and primary tumor (green) and metastatic (blue) samples. Note that normal tissue shows a dense peak at high H-score values, whereas both primary and metastatic renal cell carcinoma are characterized by low 5hmC levels. (B) Association of 5hmC and Fuhrman grade. Box plots show distribution of 5hmC H-scores in different Fuhrman grade groups. (C) Breakdown of 5hmC staining levels in Fuhrman groups. Note the shift in 5hmC H-score levels at the transition from grade ≤ 2 to ≥ 3.
(PDF)

S7 Fig Association between 5hmC levels and survival in CCRCC. **(**A) Fuhrman grade and (B) TNM stage are predictors of prognosis in the patient cohort investigated in this study. (C) 5hmC levels are not associated with cancer related mortality. (D) Box plot showing distribution of 5hmC levels stratified by disease specific mortality. Note that the numbers of patient with disease progression are low precluding a definitive statement on the association between 5hmC levels and disease progression.
(PDF)

S8 Fig Correlation between 5hmC levels and previously determined markers of the mTOR pathway. **(**A) Correlation matrix of investigated markers. Heat map shows correlation coefficient and directionality. (B, C, D) scatter plots of statistically significantly correlated markers.
(PDF)

S9 Fig DNMT1 staining in urological malignancies. **(**A) Immunolabeling of DNMT1 in normal urothelium reveals immunoreactivity in the intermediate and apical cell layer. (B) Urothelial cell carcinoma, both invasive and (C) non-invasive types, show high expression of DNMT1. (D) Renal cell carcinoma shows greatly reduced DNMT1 levels in neoplastic cells. (E) Seminoma, despite its known greatly reduced level of 5mC shows strong DNMT1 expression. Note that no direct correlation between DNMT1 and 5hmC distribution was observed.
(PDF)

S10 Fig Co-immunolabeling of 5hmC and ki67 in normal testis and seminoma. Note that in normal seminiferous tubules Sertoli cells show strong immunoreactivity for 5hmC. Seminoma shows greatly reduced 5hmC levels and interspersed ki67 positive cells (arrows).
(PDF)

S1 Table (PDF)

S2 Table (PDF)

## References

[1] Rodríguez-Paredes M, Esteller M. Cancer epigenetics reaches mainstream oncology. Nat Med. 2011 3;17(3):330–9. 10.1038/nm.2305 21386836

[2] Esteller M. Epigenetics in cancer. N Engl J Med. 2008 3 13;358(11):1148–59. 10.1056/NEJMra072067 18337604

[3] Jones PA, Baylin SB. The epigenomics of cancer. Cell. 2007 2 23;128(4):683–92. 17320506 10.1016/j.cell.2007.01.029 PMC3894624

[4] Nelson WG, De Marzo AM, Yegnasubramanian S. Epigenetic alterations in human prostate cancers. Endocrinology. 2009 9;150(9):3991–4002. 10.1210/en.2009-0573 19520778 PMC2736081

[5] Johnstone SE, Baylin SB. Stress and the epigenetic landscape: a link to the pathobiology of human diseases? Nat Rev Genet. 2010 11;11(11):806–12. 10.1038/nrg2881 20921961 PMC3148009

[6] Hemberger M, Dean W, Reik W. Epigenetic dynamics of stem cells and cell lineage commitment: digging Waddington's canal. Nat Rev Mol Cell Biol. 2009 8;10(8):526–37. 10.1038/nrm2727 19603040

[7] Kangaspeska S, Stride B, Métivier R, Polycarpou-Schwarz M, Ibberson D, Carmouche RP, et al Transient cyclical methylation of promoter DNA. Nature. 2008 3 6;452(7183):112–5. 10.1038/nature06640 18322535

[8] Aryee MJ, Liu W, Engelmann JC, Nuhn P, Gurel M, Haffner MC, et al DNA methylation alterations exhibit intraindividual stability and interindividual heterogeneity in prostate cancer metastases. Sci Transl Med. American Association for the Advancement of Science; 2013 1 23;5(169):169ra10–0. 10.1126/scitranslmed.3005211 23345608 PMC3577373

[9] Penn NW, Suwalski R, O'Riley C, Bojanowski K, Yura R. The presence of 5-hydroxymethylcytosine in animal deoxyribonucleic acid. Biochem J. 1972 2;126(4):781–90. 4538516 10.1042/bj1260781 PMC1178489

[10] Tahiliani M, Koh KP, Shen Y, Pastor WA, Bandukwala H, Brudno Y, et al Conversion of 5-methylcytosine to 5-hydroxymethylcytosine in mammalian DNA by MLL partner TET1. Science. American Association for the Advancement of Science; 2009 5 15;324(5929):930–5. 10.1126/science.1170116 19372391 PMC2715015

[11] Shen L, Zhang Y. 5-Hydroxymethylcytosine: generation, fate, and genomic distribution. Curr Opin Cell Biol. Elsevier Ltd; 2013 6 1;25(3):289–96. 10.1016/j.ceb.2013.02.017 23498661 PMC4060438

[12] Williams K, Christensen J, Helin K. DNA methylation: TET proteins-guardians of CpG islands? EMBO Rep. 2012 1;13(1):28–35. 10.1038/embor.2011.233 PMC3246258 22157888

[13] Kohli RM, Zhang Y. TET enzymes, TDG and the dynamics of DNA demethylation. Nature. 2013 10 23;502(7472):472–9. 10.1038/nature12750 24153300 PMC4046508

[14] He Y-F, Li B-Z, Li Z, Liu P, Wang Y, Tang Q, et al Tet-mediated formation of 5-carboxylcytosine and its excision by TDG in mammalian DNA. Science. American Association for the Advancement of Science; 2011 9 2;333(6047):1303–7. 10.1126/science.1210944 21817016 PMC3462231

[15] Ito S, Shen L, Dai Q, Wu SC, Collins LB, Swenberg JA, et al Tet proteins can convert 5-methylcytosine to 5-formylcytosine and 5-carboxylcytosine. Science. American Association for the Advancement of Science; 2011 9 2;333(6047):1300–3. 10.1126/science.1210597 21778364 PMC3495246

[16] Serandour AA, Avner S, Oger F, Bizot M, Percevault F, Lucchetti-Miganeh C, et al Dynamic hydroxymethylation of deoxyribonucleic acid marks differentiation-associated enhancers. Nucleic Acids Res. Oxford University Press; 2012 9 1;40(17):8255–65. 22730288 10.1093/nar/gks595 PMC3458548

[17] Guibert S, Weber M. Functions of DNA methylation and hydroxymethylation in mammalian development. Curr Top Dev Biol. Elsevier; 2013;104:47–83. 10.1016/B978-0-12-416027-9.00002-4 23587238

[18] Haffner MC, Chaux A, Meeker AK, Esopi DM, Gerber J, Pellakuru LG, et al Global 5-hydroxymethylcytosine content is significantly reduced in tissue stem/progenitor cell compartments and in human cancers. Oncotarget. 2011 8;2(8):627–37. 21896958 10.18632/oncotarget.316 PMC3248214

[19] Orr BA, Haffner MC, Nelson WG, Yegnasubramanian S, Eberhart CG. Decreased 5-hydroxymethylcytosine is associated with neural progenitor phenotype in normal brain and shorter survival in malignant glioma. Christensen BC, editor. PLoS ONE. Public Library of Science; 2012;7(7):e41036 10.1371/journal.pone.0041036 22829908 PMC3400598

[20] Pfeifer GP, Kadam S, Jin S-G. 5-hydroxymethylcytosine and its potential roles in development and cancer. Epigenetics Chromatin. BioMed Central Ltd; 2013;6(1):10 10.1186/1756-8935-6-10 23634848 PMC3645968

[21] Jin S-G, Jiang Y, Qiu R, Rauch TA, Wang Y, Schackert G, et al 5-Hydroxymethylcytosine is strongly depleted in human cancers but its levels do not correlate with IDH1 mutations. Cancer Res. American Association for Cancer Research; 2011 12 15;71(24):7360–5. 10.1158/0008-5472.CAN-11-2023 22052461 PMC3242933

[22] Haffner MC, Pellakuru LG, Ghosh S, Lotan TL, Nelson WG, De Marzo AM, et al Tight correlation of 5-hydroxymethylcytosine and Polycomb marks in health and disease. Cell Cycle. 2013 6 15;12(12):1835–41. 10.4161/cc.25010 23676216 PMC3735697

[23] Larson AR, Dresser KA, Zhan Q, Lezcano C, Woda BA, Yosufi B, et al Loss of 5-hydroxymethylcytosine correlates with increasing morphologic dysplasia in melanocytic tumors. Nature Publishing Group; 2014 1 3;:1–9. 10.1038/modpathol.2013.224 PMC4077910 24390216

[24] Lian CG, Xu Y, Ceol C, Wu F, Larson A, Dresser K, et al Loss of 5-hydroxymethylcytosine is an epigenetic hallmark of melanoma. Cell. 2012 9 14;150(6):1135–46. 10.1016/j.cell.2012.07.033 22980977 PMC3770275

[25] Kroeze LI, Aslanyan MG, van Rooij A, Koorenhof-Scheele TN, Massop M, Carell T, et al Characterization of acute myeloid leukemia based on levels of global hydroxymethylation. Blood. 2014 8 14;124(7):1110–8. 10.1182/blood-2013-08-518514 24986689

[26] Nettersheim D, Heukamp LC, Fronhoffs F, Grewe MJ, Haas N, Waha A, et al Analysis of TET expression/activity and 5mC oxidation during normal and malignant germ cell development. Deb S, editor. PLoS ONE. Public Library of Science; 2013;8(12):e82881 10.1371/journal.pone.0082881 24386123 PMC3873252

[27] Liu C, Liu L, Chen X, Shen J, Shan J, Xu Y, et al Decrease of 5-hydroxymethylcytosine is associated with progression of hepatocellular carcinoma through downregulation of TET1. Guan X-Y, editor. PLoS ONE. Public Library of Science; 2013;8(5):e62828 10.1371/journal.pone.0062828 23671639 PMC3650038

[28] Fedor HL, De Marzo AM. Practical methods for tissue microarray construction. Methods Mol Med. 2005;103:89–101. 15542899 10.1385/1-59259-780-7:089

[29] Netto GJ, Nakai Y, Nakayama M, Jadallah S, Toubaji A, Nonomura N, et al Global DNA hypomethylation in intratubular germ cell neoplasia and seminoma, but not in nonseminomatous male germ cell tumors. Mod Pathol. 2008 11;21(11):1337–44. 10.1038/modpathol.2008.127 18622385 PMC4086525

[30] Schultz L, Chaux A, Albadine R, Hicks J, Kim JJ, De Marzo AM, et al Immunoexpression status and prognostic value of mTOR and hypoxia-induced pathway members in primary and metastatic clear cell renal cell carcinomas. Am J Surg Pathol. 2011 10;35(10):1549–56. 10.1097/PAS.0b013e31822895e5 21881486 PMC3505672

[31] Hahn MA, Qiu R, Wu X, Li AX, Zhang H, Wang J, et al Dynamics of 5-hydroxymethylcytosine and chromatin marks in Mammalian neurogenesis. CellReports. 2013 2 21;3(2):291–300. 10.1016/j.celrep.2013.01.011 PMC3582786 23403289

[32] Iqbal K, Jin S-G, Pfeifer GP, Szabó PE. Reprogramming of the paternal genome upon fertilization involves genome-wide oxidation of 5-methylcytosine. Proc Natl Acad Sci USA. 2011 3 1;108(9):3642–7. 10.1073/pnas.1014033108 21321204 PMC3048122

[33] Globisch D, Münzel M, Müller M, Michalakis S, Wagner M, Koch S, et al Tissue distribution of 5-hydroxymethylcytosine and search for active demethylation intermediates. Croft AK, editor. PLoS ONE. 2010;5(12):e15367 10.1371/journal.pone.0015367 21203455 PMC3009720

[34] Delhommeau F, Dupont S, Valle Della V, James C, Trannoy S, Massé A, et al Mutation in TET2 in myeloid cancers. N Engl J Med. 2009 5 28;360(22):2289–301. 10.1056/NEJMoa0810069 19474426

[35] Yang H, Liu Y, Bai F, Zhang J-Y, Ma S-H, Liu J, et al Tumor development is associated with decrease of TET gene expression and 5-methylcytosine hydroxylation. Oncogene. Nature Publishing Group; 2012 3 5;32(5):663–9. 10.1038/onc.2012.67 22391558 PMC3897214

[36] Figueroa ME, Abdel-Wahab O, Lu C, Ward PS, Patel J, Shih A, et al Leukemic IDH1 and IDH2 mutations result in a hypermethylation phenotype, disrupt TET2 function, and impair hematopoietic differentiation. Cancer Cell. 2010 12 14;18(6):553–67. 10.1016/j.ccr.2010.11.015 21130701 PMC4105845

[37] Müller T, Gessi M, Waha A, Isselstein LJ, Luxen D, Freihoff D, et al Nuclear exclusion of TET1 is associated with loss of 5-hydroxymethylcytosine in IDH1 wild-type gliomas. The American Journal of Pathology. 2012 8;181(2):675–83. 10.1016/j.ajpath.2012.04.017 22688054

[38] Shim E-H, Livi CB, Rakheja D, Tan J, Benson D, Parekh V, et al L-2-Hydroxyglutarate: An Epigenetic Modifier and Putative Oncometabolite in Renal Cancer. Cancer Discovery. 2014 9 2. 10.1158/2159-8290.CD-13-0696 PMC4286872 25182153

[39] Chen C-C, Wang K-Y, Shen C-KJ. The mammalian de novo DNA methyltransferases DNMT3A and DNMT3B are also DNA 5-hydroxymethylcytosine dehydroxymethylases. J Biol Chem. American Society for Biochemistry and Molecular Biology; 2012 9 28;287(40):33116–21. 22898819 10.1074/jbc.C112.406975 PMC3460417

[40] Robinson CM, Ohh M. The multifaceted von Hippel-Lindau tumour suppressor protein. FEBS Lett. 2014 8 19;588(16):2704–11. 10.1016/j.febslet.2014.02.026 24583008

[41] Smith ZD, Meissner A. The simplest explanation: passive DNA demethylation in PGCs. EMBO J. 2013 2 6;32(3):318–21. 10.1038/emboj.2012.349 23299938 PMC3567498

[42] Inoue A, Zhang Y. Replication-dependent loss of 5-hydroxymethylcytosine in mouse preimplantation embryos. Science. American Association for the Advancement of Science; 2011 10 14;334(6053):194–4. 10.1126/science.1212483 21940858 PMC3799877

[43] Nestor CE, Ottaviano R, Reddington J, Sproul D, Reinhardt D, Dunican D, et al Tissue type is a major modifier of the 5-hydroxymethylcytosine content of human genes. Genome Res. Cold Spring Harbor Lab; 2012 3;22(3):467–77. 10.1101/gr.126417.111 22106369 PMC3290782

[44] Haffner MC, Pellakuru LG, Ghosh S, Lotan TL, Nelson WG, De Marzo AM, et al Tight correlation of 5-hydroxymethylcytosine and Polycomb marks in health and disease. Cell Cycle. Landes Bioscience; 2013 6 15;12(12):1835–41. 10.4161/cc.25010 23676216 PMC3735697

[45] Kristensen DG, Nielsen JE, rgensen AJO, k NESA, Meyts ER-D, Almstrup K. Evidence that active demethylation mechanisms maintain the genome of carcinoma in situ cells hypomethylated in the adult testis. Nature Publishing Group; 2013 11 28;:1–11. 10.1038/bjc.2013.727 PMC3915112 24292451

[46] Plongthongkum N, Diep DH, Zhang K. Advances in the profiling of DNA modifications: cytosine methylation and beyond. Nat Rev Genet. 2014 10;15(10):647–61. 10.1038/nrg3772 25159599

