# Supplementary material for: Global 5-Hydroxymethylcytosine Levels Are Profoundly Reduced in Multiple Genitourinary Malignancies
Source: PLoS One. 2016 Jan 19;11(1):e0146302. doi: 10.1371/journal.pone.0146302 (PMC4718593; doi:10.1371/journal.pone.0146302)
Supplement: S1 Fig — (PDF) [file pone.0146302.s002.pdf]

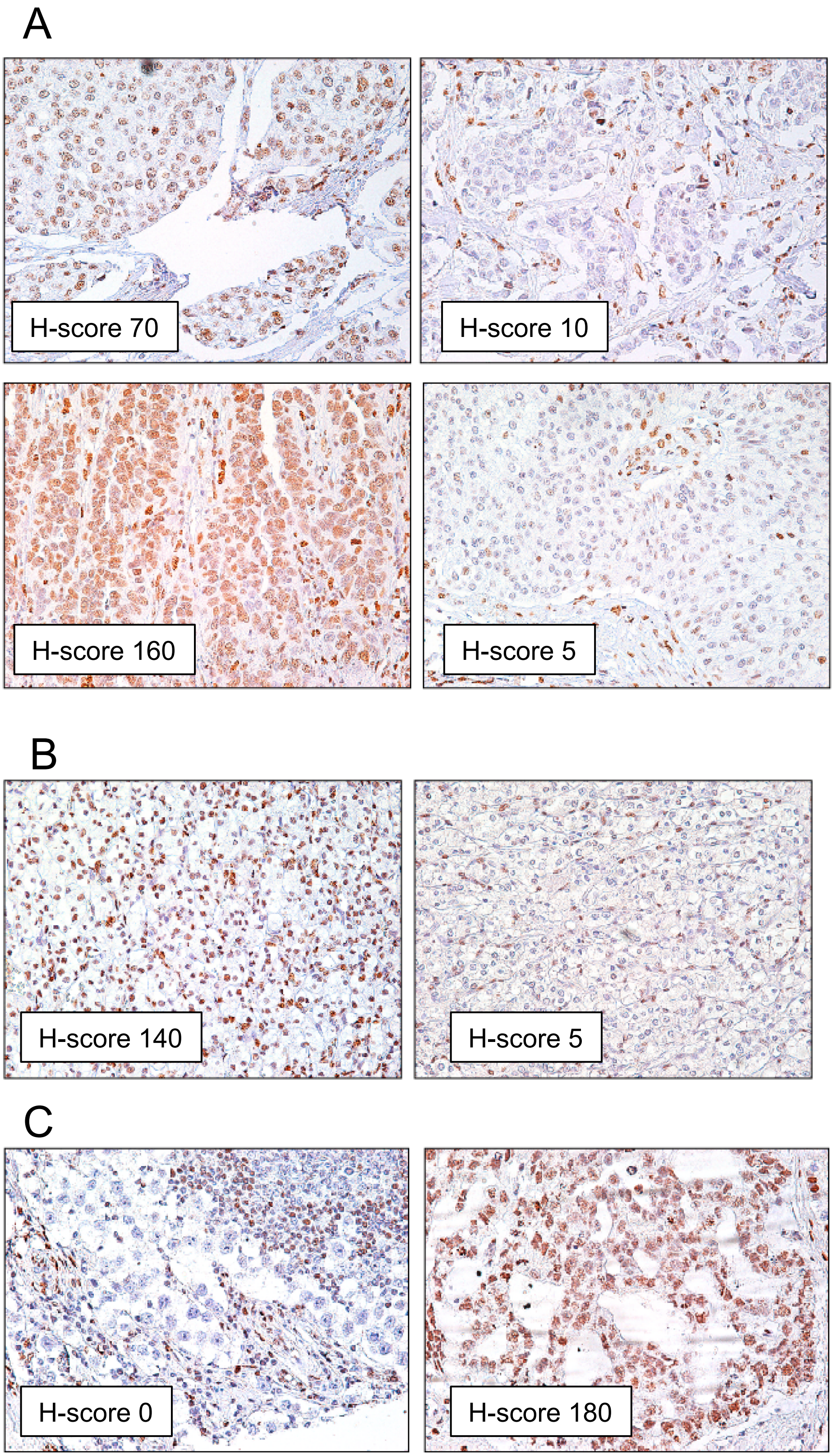

S1 Fig. Representative images and H-scores of (A) urothelial carcinoma, (B) clear cell renal cell carcinoma and (C) testicular germ cell tumors stained for 5hmC.
