# Supplementary material for: Global 5-Hydroxymethylcytosine Levels Are Profoundly Reduced in Multiple Genitourinary Malignancies
Source: PLoS One. 2016 Jan 19;11(1):e0146302. doi: 10.1371/journal.pone.0146302 (PMC4718593; doi:10.1371/journal.pone.0146302)
Supplement: S2 Fig — (A) Co-immunolabeling of 5hmC (red) and ki67 (green) in normal urothelium shows low ki67 labeling (mostly localized in the intermediate cell layer) and a gradual increase of 5hmC with increased distance from the basal cell layer. (B) In urothelial carcinoma 5hmC levels are uniformly reduced. Ki67 labeling is present in a large fraction of cells (arrows). No difference in 5hmC immunoreactivity is detected between ki67 positive and ki67 negative cells. (PDF) [file pone.0146302.s003.pdf]

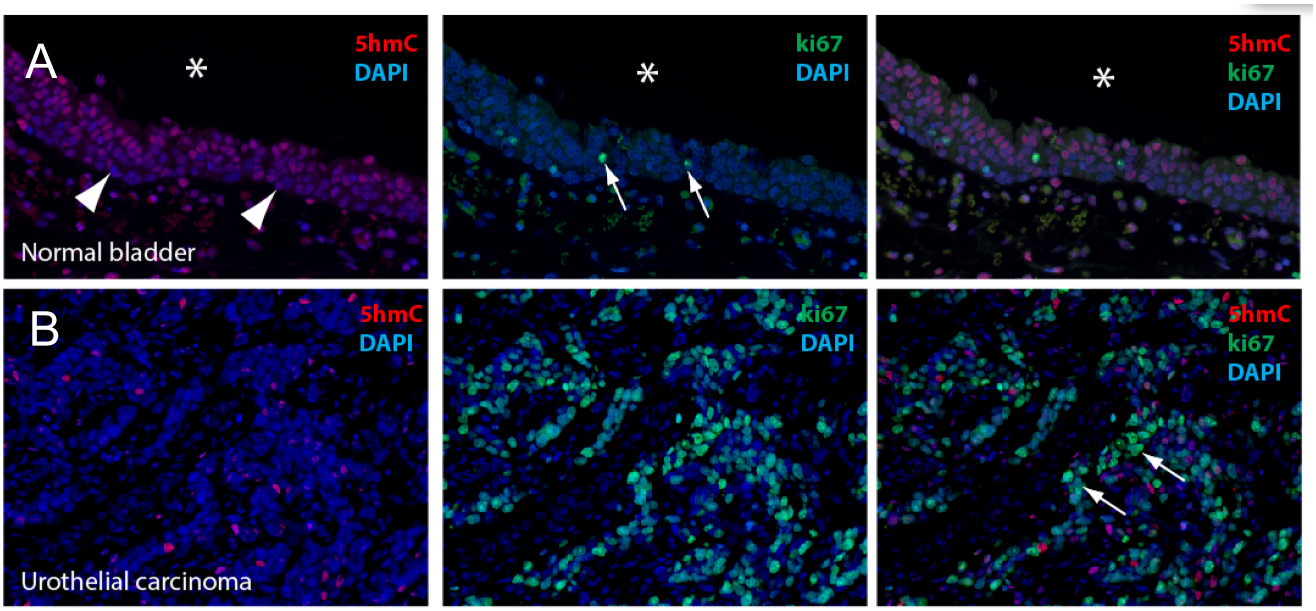

S2 Fig. Co-immunolabeling of 5hmC and ki67 in normal bladder and urothelial carcinoma of the bladder. (A) Co-immunolabeling of 5hmC (red) and ki67 (green) in normal urothelium shows low ki67 labeling (mostly localized in the intermediate cell layer) and a gradual increase of 5hmC with increased distance from the basal cell layer. (B) In urothelial carcinoma 5hmC levels are uniformly reduced. Ki67 labeling is present in a large fraction of cells (arrows). No difference in 5hmC immunoreactivity is detected between ki67 positive and ki67 negative cells.
