# Supplementary material for: Global 5-Hydroxymethylcytosine Levels Are Profoundly Reduced in Multiple Genitourinary Malignancies
Source: PLoS One. 2016 Jan 19;11(1):e0146302. doi: 10.1371/journal.pone.0146302 (PMC4718593; doi:10.1371/journal.pone.0146302)
Supplement: S3 Fig — (A) Box plot shows 5hmC H-score distribution of tumor and normal urothelium. (B) H-score distribution in invasive and non-invasive urothelial cell carcinoma. (C) Representative micrographs of invasive urothelial carcinoma of the baldder stained for 5hmC and p53. (D) Representative micrographs of non-invasive urothelial carcinoma of the baldder stained for 5hmC and p53. Note that Invasive carcinoma show nuclear accumulation of p53 suggestive of mutant TP53. 5hmC levels are not different between invasive and non-invasive carcinoma. (PDF) [file pone.0146302.s004.pdf]

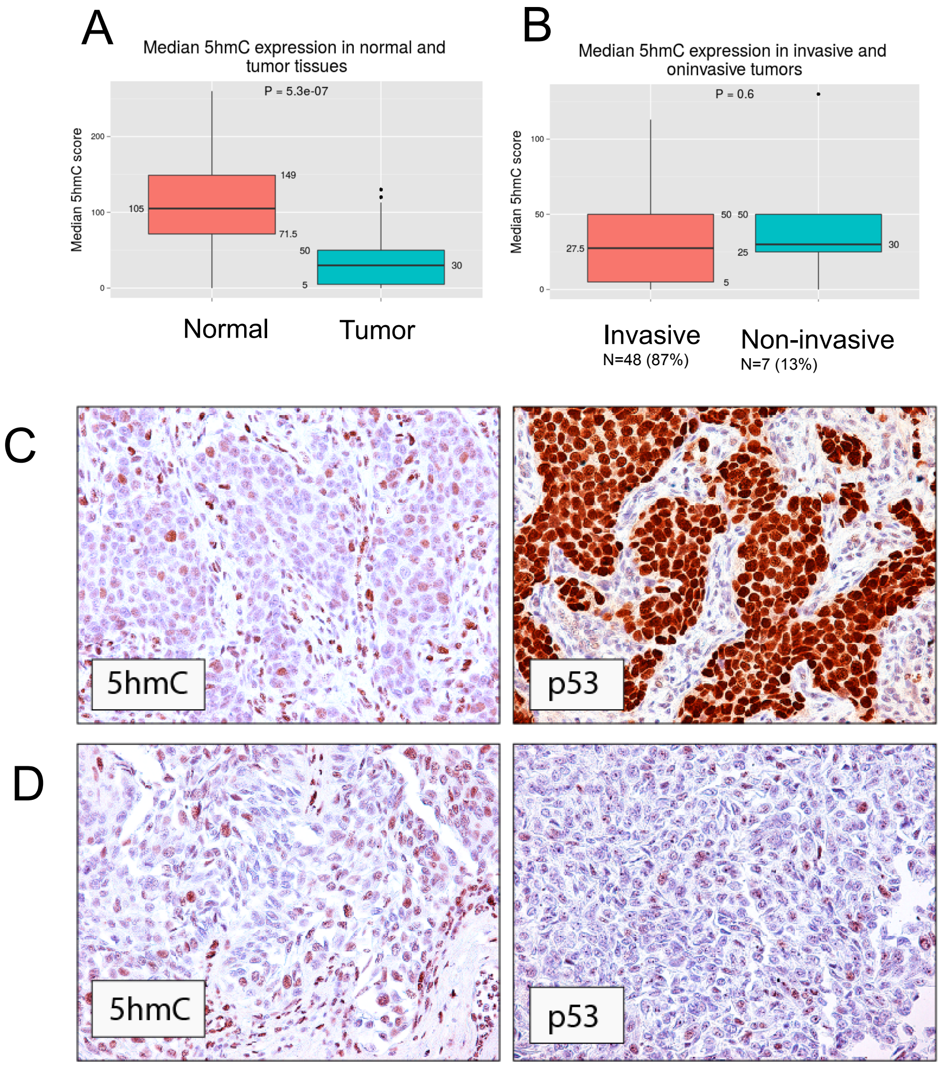

S3 Fig. 5hmC levels in invasive and superficial urothelial cell carcinoma of the bladder. (A) Box plot shows 5hmC H-score distribution of tumor and normal urothelium. (B) H-score distribution in invasive and non-invasive urothelial cell carcinoma. (C) Representative micrographs of invasive urothelial carcinoma of the baldder stained for 5hmC and p53. (D) Representative micrographs of non-invasive urothelial carcinoma of the baldder stained for 5hmC and p53. Note that Invasive carcinoma show nuclear accumulation of p53 suggestive of mutant *TP53*. 5hmC levels are not different between invasive and non-invasive carcinoma.
