# Supplementary material for: Global 5-Hydroxymethylcytosine Levels Are Profoundly Reduced in Multiple Genitourinary Malignancies
Source: PLoS One. 2016 Jan 19;11(1):e0146302. doi: 10.1371/journal.pone.0146302 (PMC4718593; doi:10.1371/journal.pone.0146302)
Supplement: S4 Fig — (A) Box plot showing median H-score distribution between normal urothelium and urothelial cell carcinoma. (B) Box plot show 5hmC level differences between specimens with and without progression to metastasis. (C) Box plot showing 5hmC levels in lesions with and without synchronous lymph node metastasis. (D) Box plot showing 5hmC levels between specimens with and without tumor progression. (E) Box plot showing 5hmC levels based on cancer specific survival. (F) Lymph node metastasis at the time of surgery is a strong predictor for disease specific and overall mortality. (G) 5hmC levels are not associated with cancer related mortality. P-values for boxplot comparisons by Wilcoxon rank test. P-values from Kaplan-Meier analysis are log-rank test derived. (PDF) [file pone.0146302.s005.pdf]

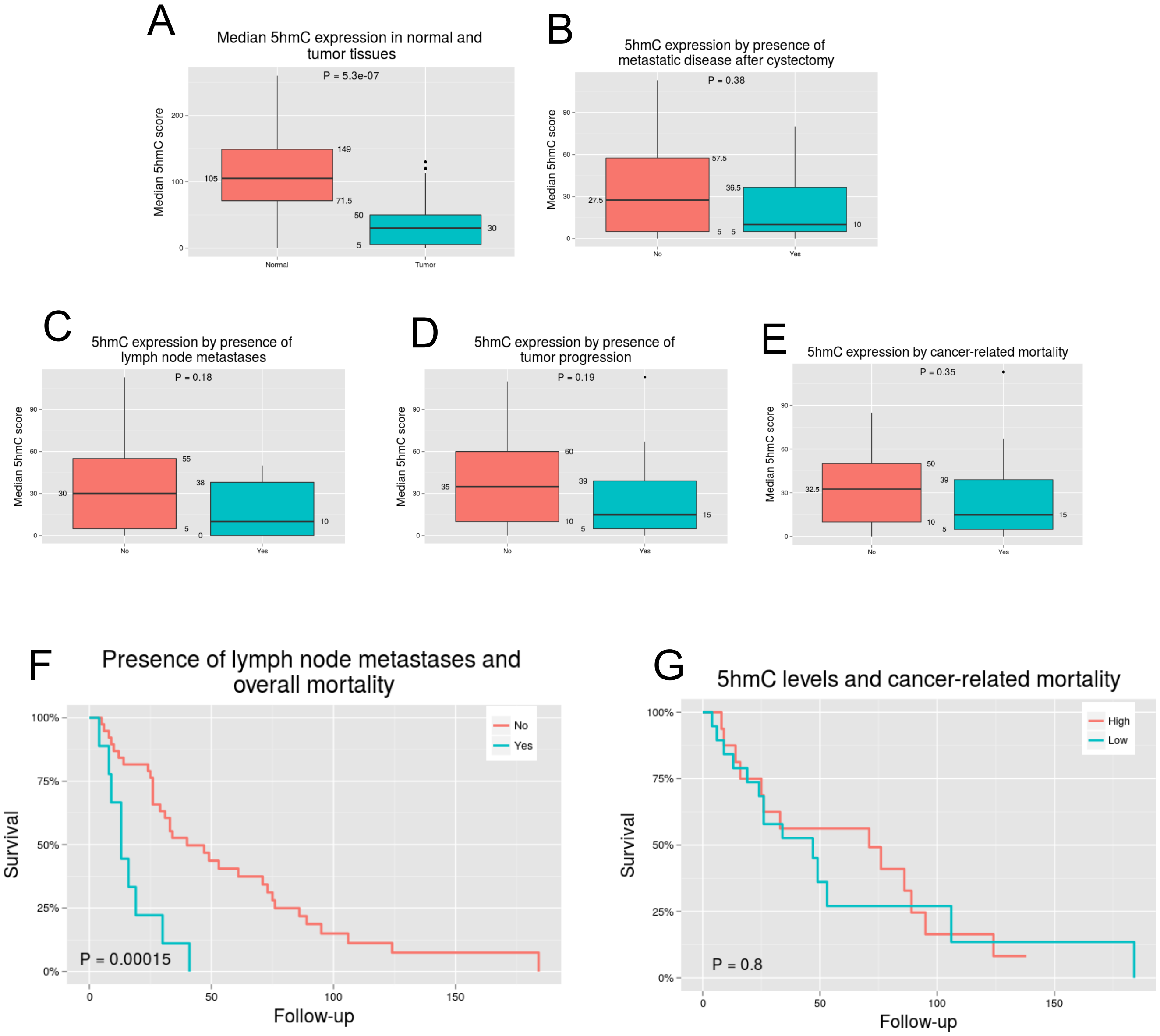

S4 Fig. Association of global 5hmC levels with clinicopathological characteristics in urothelial carcinoma. (A) Box plot showing median H-score distribution between normal urothelium and urothelial cell carcinoma. (B) Box plot show 5hmC level differences between specimens with and without progression to metastasis. (C) Box plot showing 5hmC levels in lesions with and without synchronous lymph node metastasis. (D) Box plot showing 5hmC levels between specimens with and without tumor progression. (E) Box plot showing 5hmC levels based on cancer specific survival. (F) Lymph node metastasis at the time of surgery is a strong predictor for disease specific and overall mortality. (G) 5hmC levels are not associated with cancer related mortality. P-values for boxplot comparisons by Wilcoxon rank test. P-values from Kaplan-Meier analysis are log-rank test derived.
