# Supplementary material for: Global 5-Hydroxymethylcytosine Levels Are Profoundly Reduced in Multiple Genitourinary Malignancies
Source: PLoS One. 2016 Jan 19;11(1):e0146302. doi: 10.1371/journal.pone.0146302 (PMC4718593; doi:10.1371/journal.pone.0146302)
Supplement: S5 Fig — Note that 5hmC levels are high in normal kidney tissue and greatly reduced in renal cell carcinoma. Sporadic ki67 positive cells (stained in green) can be found in carcinoma. Note that no direct association between ki67 positive cells and 5hmC staining was observed. (PDF) [file pone.0146302.s006.pdf]

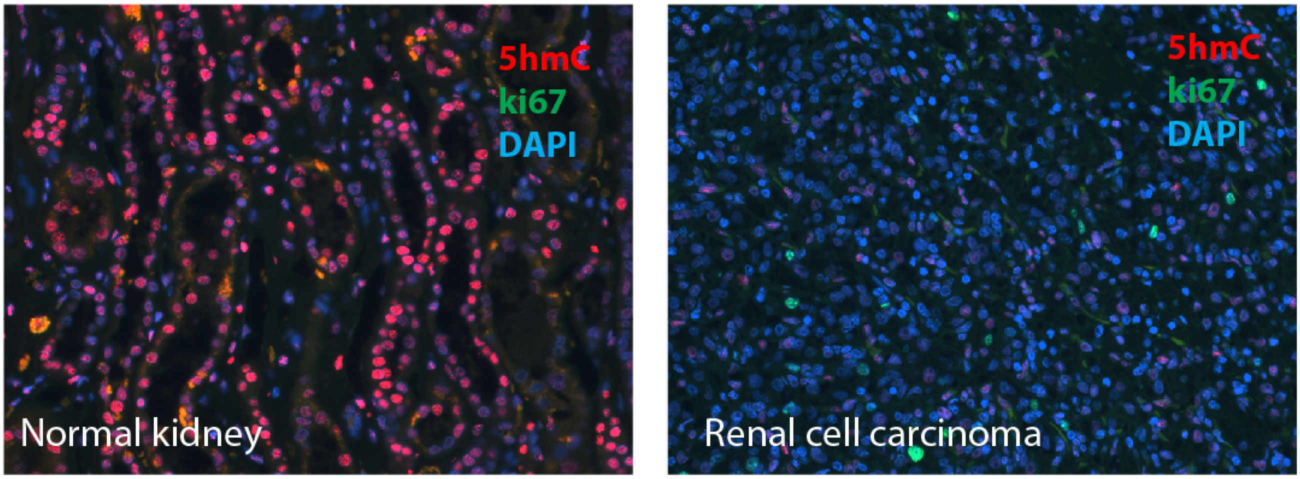

S5 Fig. Co-immunolabeling of 5hmC and ki67 in (A) normal kidney and (B) clear cell renal cell carcinoma. Note that 5hmC levels are high in normal kidney tissue and greatly reduced in renal cell carcinoma. Sporadic ki67 positive cells (stained in green) can be found in carcinoma. Note that no direct association between ki67 positive cells and 5hmC staining was observed.
