# Supplementary material for: Global 5-Hydroxymethylcytosine Levels Are Profoundly Reduced in Multiple Genitourinary Malignancies
Source: PLoS One. 2016 Jan 19;11(1):e0146302. doi: 10.1371/journal.pone.0146302 (PMC4718593; doi:10.1371/journal.pone.0146302)
Supplement: S6 Fig — (A) Density plot shows distribution of 5hmC levels in normal tissue (red) and primary tumor (green) and metastatic (blue) samples. Note that normal tissue shows a dense peak at high H-score values, whereas both primary and metastatic renal cell carcinoma are characterized by low 5hmC levels. (B) Association of 5hmC and Fuhrman grade. Box plots show distribution of 5hmC H-scores in different Fuhrman grade groups. (C) Breakdown of 5hmC staining levels in Fuhrman groups. Note the shift in 5hmC H-score levels at the transition from grade ≤ 2 to ≥ 3. (PDF) [file pone.0146302.s007.pdf]

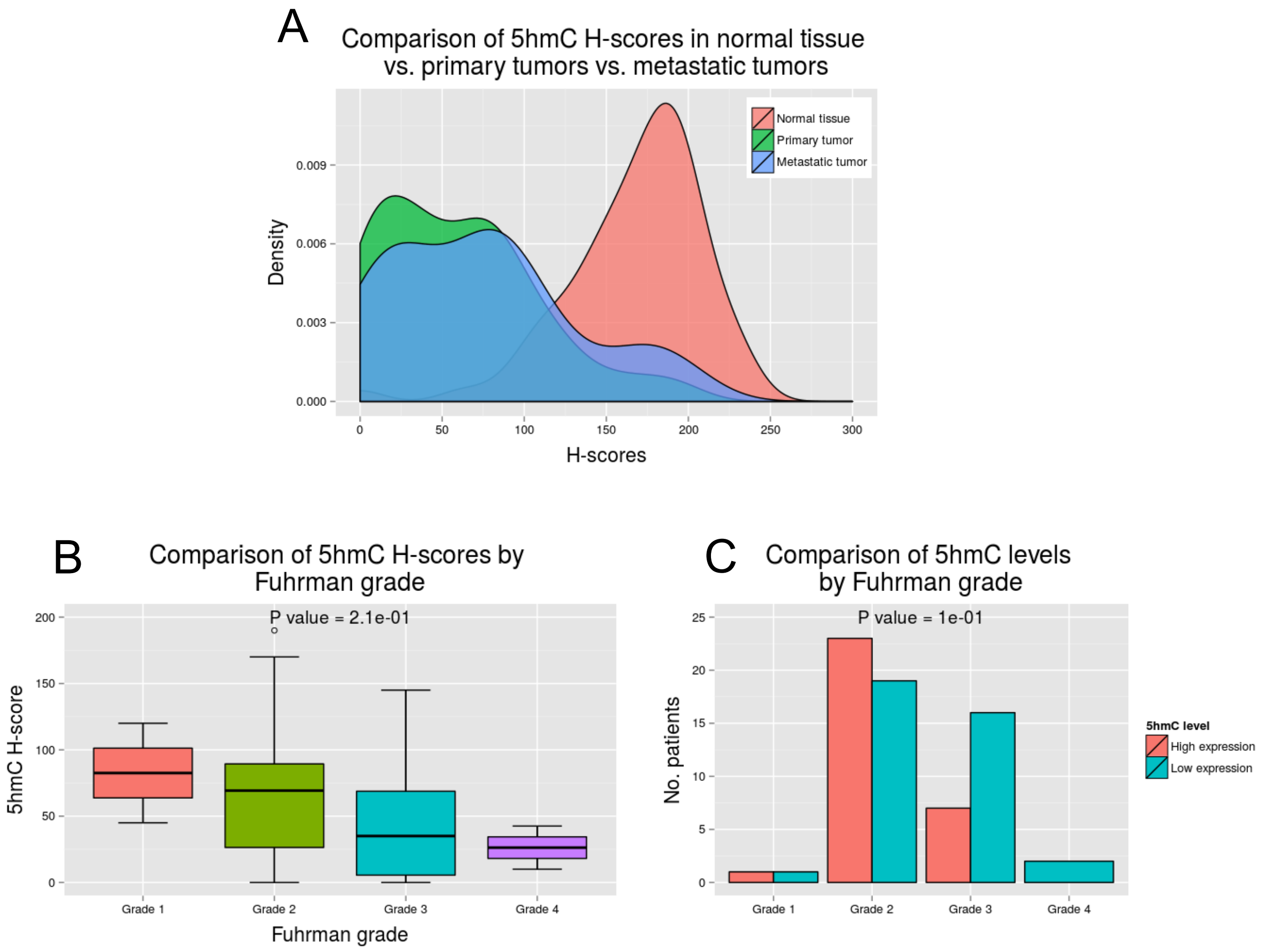

S6 Fig. 5hmC levels in normal kidney, primary renal cell carcinoma and metastatic renal cell carcinoma. (A) Density plot shows distribution of 5hmC levels in normal tissue (red) and primary tumor (green) and metastatic (blue) samples. Note that normal tissue shows a dense peak at high H-score values, whereas both primary and metastatic renal cell carcinoma are characterized by low 5hmC levels. (B) Association of 5hmC and Fuhrman grade. Box plots show distribution of 5hmC H-scores in different Fuhrman grade groups. (C) Breakdown of 5hmC staining levels in Fuhrman groups. Note the shift in 5hmC H-score levels at the transition from grade ≤ 2 to ≥ 3.
