# Supplementary material for: Global 5-Hydroxymethylcytosine Levels Are Profoundly Reduced in Multiple Genitourinary Malignancies
Source: PLoS One. 2016 Jan 19;11(1):e0146302. doi: 10.1371/journal.pone.0146302 (PMC4718593; doi:10.1371/journal.pone.0146302)
Supplement: S7 Fig — (A) Fuhrman grade and (B) TNM stage are predictors of prognosis in the patient cohort investigated in this study. (C) 5hmC levels are not associated with cancer related mortality. (D) Box plot showing distribution of 5hmC levels stratified by disease specific mortality. Note that the numbers of patient with disease progression are low precluding a definitive statement on the association between 5hmC levels and disease progression. (PDF) [file pone.0146302.s008.pdf]

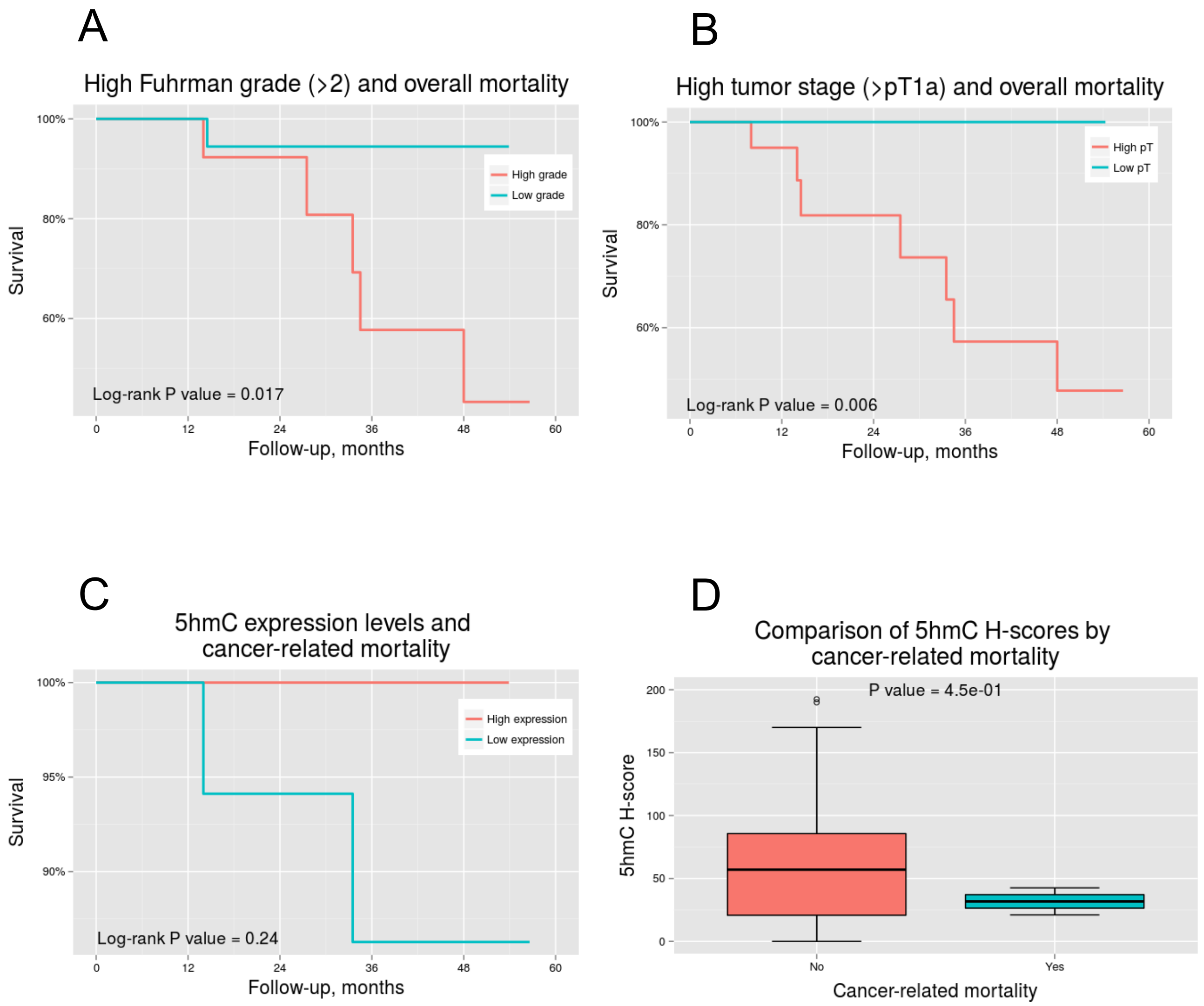

S7 Fig. Association between 5hmC levels and survival in CCRCC. (A) Fuhrman grade and (B) TNM stage are predictors of prognosis in the patient cohort investigated in this study. (C) 5hmC levels are not associated with cancer related mortality. (D) Box plot showing distribution of 5hmC levels stratified by disease specific mortality. Note that the numbers of patient with disease progression are low precluding a definitive statement on the association between 5hmC levels and disease progression.
