# Supplementary material for: Global 5-Hydroxymethylcytosine Levels Are Profoundly Reduced in Multiple Genitourinary Malignancies
Source: PLoS One. 2016 Jan 19;11(1):e0146302. doi: 10.1371/journal.pone.0146302 (PMC4718593; doi:10.1371/journal.pone.0146302)
Supplement: S8 Fig — (A) Correlation matrix of investigated markers. Heat map shows correlation coefficient and directionality. (B, C, D) scatter plots of statistically significantly correlated markers. (PDF) [file pone.0146302.s009.pdf]

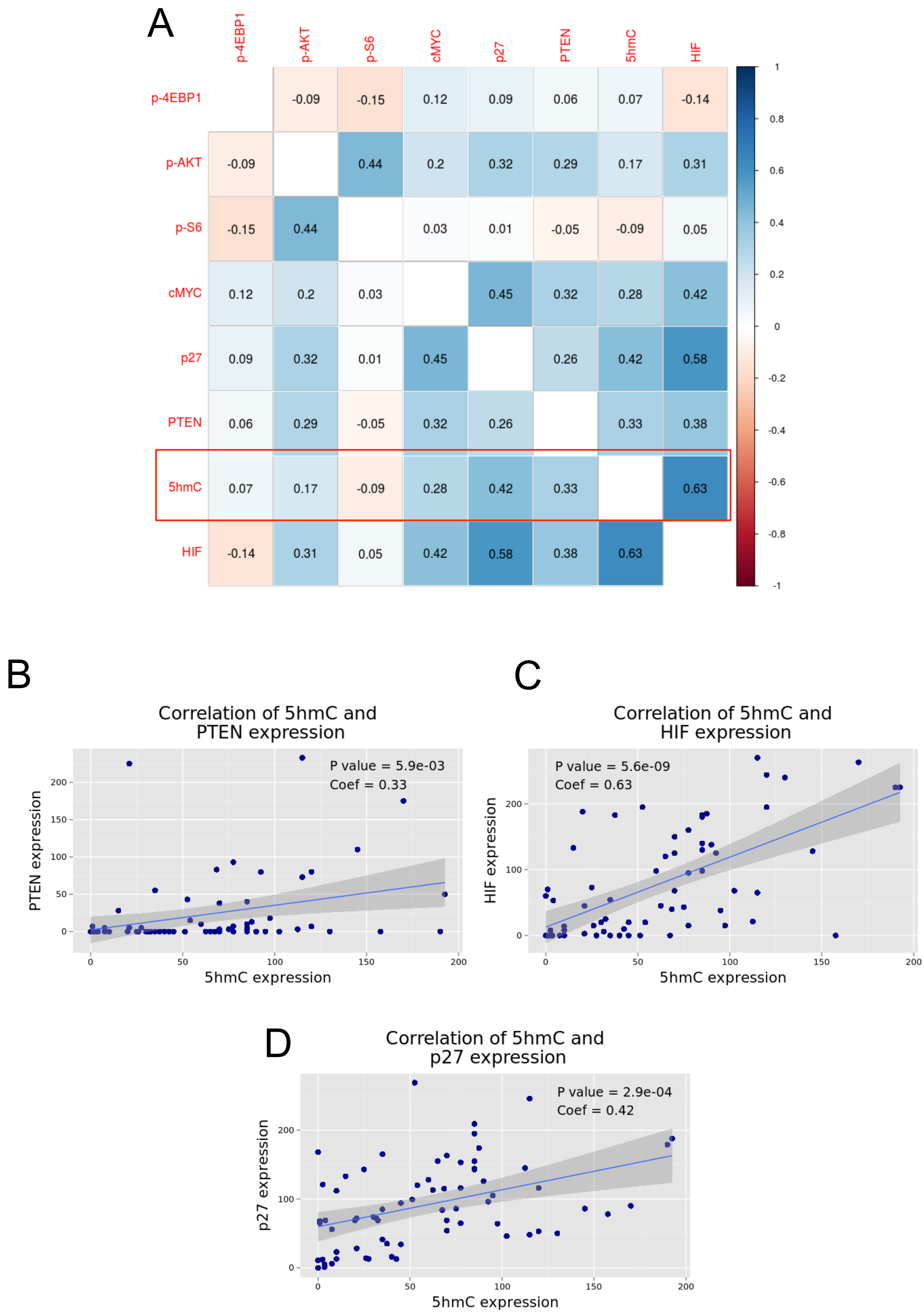

S8 Fig. Correlation between 5hmC levels and previously determined markers of the mTOR pathway. **(**A) Correlation matrix of investigated markers. Heat map shows correlation coefficient and directionality. (B, C, D) scatter plots of statistically significantly correlated markers.
