# Supplementary material for: Global 5-Hydroxymethylcytosine Levels Are Profoundly Reduced in Multiple Genitourinary Malignancies
Source: PLoS One. 2016 Jan 19;11(1):e0146302. doi: 10.1371/journal.pone.0146302 (PMC4718593; doi:10.1371/journal.pone.0146302)
Supplement: S9 Fig — (A) Immunolabeling of DNMT1 in normal urothelium reveals immunoreactivity in the intermediate and apical cell layer. (B) Urothelial cell carcinoma, both invasive and (C) non-invasive types, show high expression of DNMT1. (D) Renal cell carcinoma shows greatly reduced DNMT1 levels in neoplastic cells. (E) Seminoma, despite its known greatly reduced level of 5mC shows strong DNMT1 expression. Note that no direct correlation between DNMT1 and 5hmC distribution was observed. (PDF) [file pone.0146302.s010.pdf]

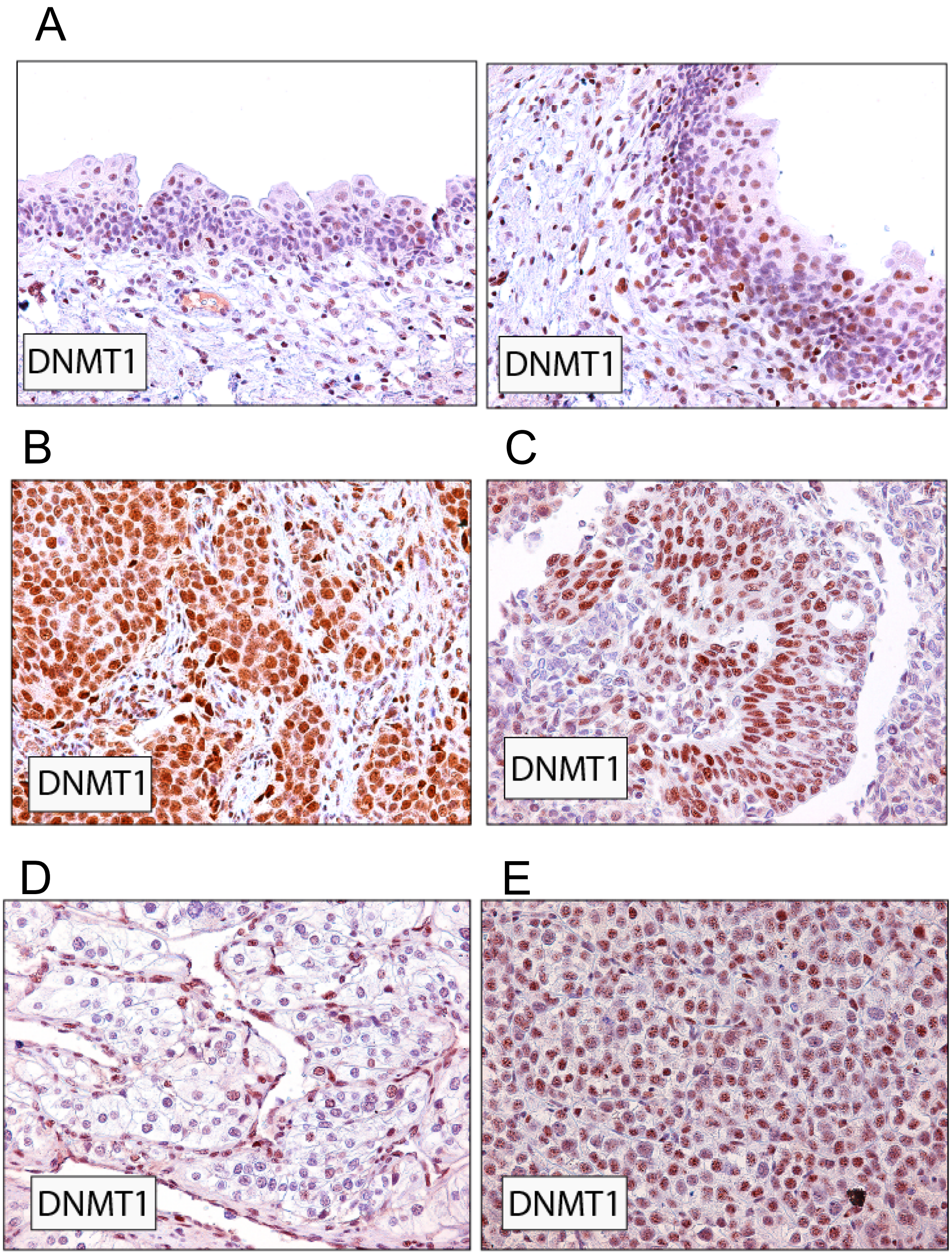

S9 Fig. DNMT1 staining in urological malignancies. (A) Immunolabeling of DNMT1 in normal urothelium reveals immunoreactivity in the intermediate and apical cell layer. (B) Urothelial cell carcinoma, both invasive and (C) non-invasive types, show high expression of DNMT1. (D) Renal cell carcinoma shows greatly reduced DNMT1 levels in neoplastic cells. (E) Seminoma, despite its known greatly reduced level of 5mC shows strong DNMT1 expression. Note that no direct correlation between DNMT1 and 5hmC distribution was observed.
