# Supplementary material for: Global 5-Hydroxymethylcytosine Levels Are Profoundly Reduced in Multiple Genitourinary Malignancies
Source: PLoS One. 2016 Jan 19;11(1):e0146302. doi: 10.1371/journal.pone.0146302 (PMC4718593; doi:10.1371/journal.pone.0146302)
Supplement: S10 Fig — Note that in normal seminiferous tubules Sertoli cells show strong immunoreactivity for 5hmC. Seminoma shows greatly reduced 5hmC levels and interspersed ki67 positive cells (arrows). (PDF) [file pone.0146302.s011.pdf]

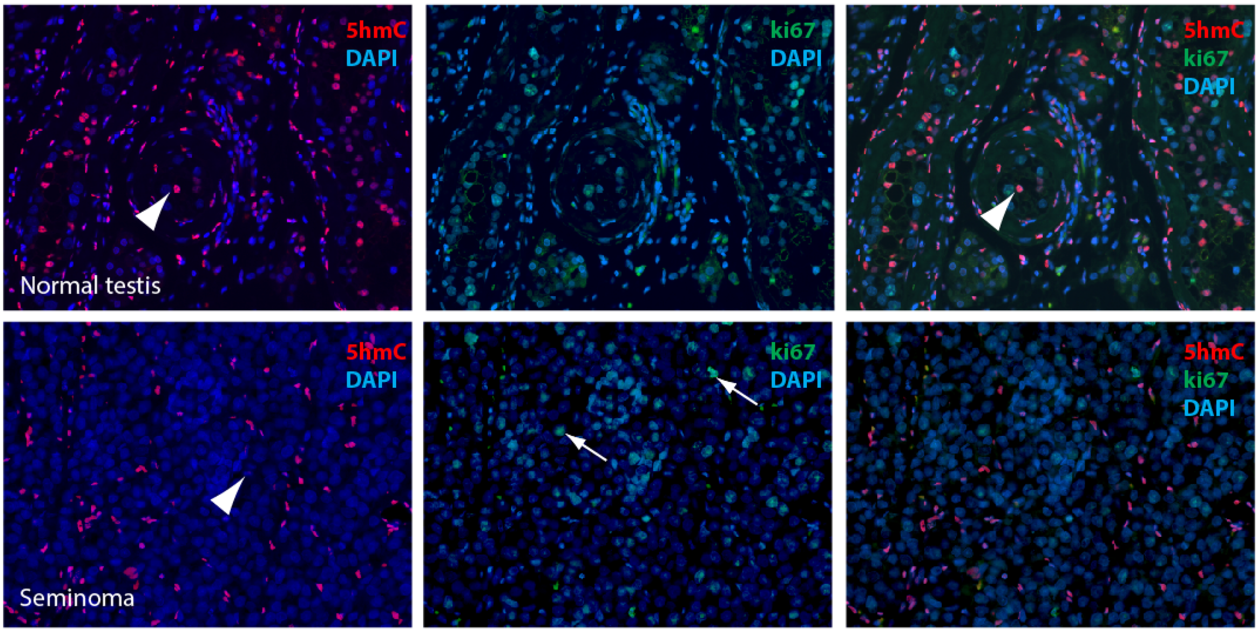

Supplementary Figure 10. Co-immunolabeling of 5hmC and ki67 in normal testis and seminoma. Note that in normal seminiferous tubules Sertoli cells show strong immunoreactivity for 5hmC. Seminoma shows greatly reduced 5hmC levels and interspersed ki67 positive cells (arrows).
