# Supplementary material for: Global 5-Hydroxymethylcytosine Levels Are Profoundly Reduced in Multiple Genitourinary Malignancies
Source: PLoS One. 2016 Jan 19;11(1):e0146302. doi: 10.1371/journal.pone.0146302 (PMC4718593; doi:10.1371/journal.pone.0146302)
Supplement: S1 Table — (PDF) [file pone.0146302.s012.pdf]

S1 Table . Cox regression for Cancer-related mortality in bladder cancer cohort.

Variables	HR	Lower 95% CI	Upper 95% CI	P value
Age group: Older vs. Younger	**0.97**	0.56	1.69	*0.92*
Sex	**2.75**	0.82	9.19	*0.062*
Race	**1.7**	0.4	7.24	*0.44*
Neoadjuvant radiotherapy	**0.55**	0.074	4.12	*0.52*
Neoadjuvant chemotherapy	**1.18**	0.35	4	*0.79*
Intravesical chemotherapy	**0.85**	0.35	2.06	*0.71*
Adjuvant radiotherapy	**0.24**	0.031	1.84	*0.089*
Adjuvant chemotherapy	**1.83**	0.84	4	*0.13*
Early complications	**1.17**	0.51	2.69	*0.71*
Late complications	**0.67**	0.31	1.46	*0.31*
Lymph node metastasis	**6.18**	2.13	17.92	*0.0024*
Distant metastasis	**2.2**	0.87	5.57	*0.096*
Lymphovascular invasion	**0.82**	0.36	1.9	*0.64*
Muscle-invasive disease	**0.52**	0.15	1.8	*0.34*
High 5hmC expression (upper median)	**1.11**	0.51	2.41	*0.8*
High 5hmC expression (upper tertile)	**1.71**	0.68	4.33	*0.23*
