# Supplementary material for: Global 5-Hydroxymethylcytosine Levels Are Profoundly Reduced in Multiple Genitourinary Malignancies
Source: PLoS One. 2016 Jan 19;11(1):e0146302. doi: 10.1371/journal.pone.0146302 (PMC4718593; doi:10.1371/journal.pone.0146302)
Supplement: S2 Table — (PDF) [file pone.0146302.s013.pdf]

S2 Table.

Cox regression: Cancer-related mortality*

Variables	P value
Age group: Above vs. below median	0.15
Sex	0.2
Race	0.77
Tumor size: Above vs. below median	0.09
High pT stage (>pT1a)	0.09
High Fuhrman grade (>2)	0.053
Tumor multifocality	0.4
Invasion of surgical margins	0.55
Tumor progression	0.0016
High 5hmC expression (upper median)	0.15
High 5hmC expression (upper tertile)	0.33
